# Ordered Mesoporous Carbon as Adsorbent for the Removal of a Triphenylmethane Dye from Its Aqueous Solutions

**DOI:** 10.3390/molecules29174100

**Published:** 2024-08-29

**Authors:** Bharti Gaur, Jyoti Mittal, Hadi Hassan, Alok Mittal, Richard T. Baker

**Affiliations:** 1 Department of Chemistry, Maulana Azad National Institute of Technology, Bhopal 462 003, India; gourbharti22@gmail.com (B.G.);; 2 School of Chemistry, University of St. Andrews, St. Andrews, Fife KY16 9ST, UK; hh204@st-andrews.ac.uk

**Keywords:** adsorption, crystal violet, ordered mesoporous carbon, isotherm, kinetics

## Abstract

A nanostructured material, ordered mesoporous carbon (OMC), was synthesised in metal- and halide-free form and its use for the sequestration of crystal violet, a hazardous triphenylmethane dye, is reported for the first time. The OMC material is characterised using scanning transmission electron microscopy with energy-dispersive spectroscopy for chemical analysis, by Fourier-transform infrared spectroscopy, and by nitrogen gas physisorption. The ideal conditions for the uptake of crystal violet dye were determined in batch experiments covering the standard parameters: pH, concentration, contact time, and adsorbent dosage. Experimental data are validated by applying Langmuir, Freundlich, Dubinin–Radushkevich, and Temkin isotherms. The thermodynamic parameters, ΔH°, ΔG°, and ΔS°, are calculated and it has been found that the adsorption process is spontaneous and endothermic with increasing disorder. An in-depth analysis of the kinetics of the adsorption process, order of the reaction and corresponding values of the rate constants was performed. The adsorption of crystal violet over OMC has been found to follow pseudo-second-order kinetics through a film diffusion process at all temperatures studied. Continuous flow column operations were performed using fixed bed adsorption. Parameters including percentage saturation of the OMC bed are evaluated. The exhausted column was regenerated through a desorption process and column efficiency was determined.

## 1. Introduction

Water contamination is a widespread problem that requires constant efforts to conserve and maintain the quality of this essential resource for generations to come. Polluting substances enter natural water damaging life forms, ecosystems and human health. Metals, dyes, non-biodegradable, hazardous, and toxic compounds, radioactive contaminants and pharmaceuticals are the most serious chemical pollutants entering the hydrosphere. Organic dyes—which usually originate from the textile, food, pharmaceuticals, and plastic industries—contribute a significant proportion of these hazardous chemicals [1,2,3]. Reports clearly indicate that—exacerbated by the faulty processing and dying procedures used by these industries—around 40,000 to 50,000 tonnes of dyes enter into water systems on a daily basis [4,5]. The presence of coloured, organic, non-biodegradable dyes degrades the oxygenation potential of water bodies and blocks sunlight, so disturbing the bioactivity of aquatic life and photosynthesis in aquatic plants and other species [6].

**Figure 1:**
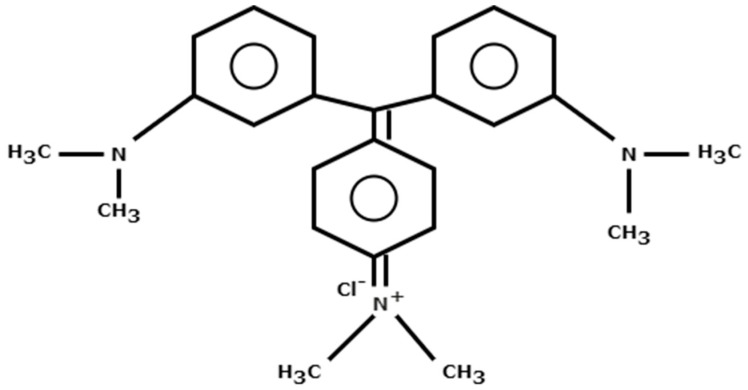
Chemical structure of the dye, crystal violet.

Crystal violet (Figure 1) is a triphenylmethane dye that is water soluble. It is additionally known as methyl violet 10B, gentian violet, or hexamethyl pararosaniline chloride, and its IUPAC name is 4-Bis[4-(dimethylamino)phenyl]methylidene-N,N-dimethylcyclohexa-2,5-dien-1-iminium chloride, chemical formula, C_25_H_30_ClN_3_ (molecular weight 408 g/mol). Some of the physical properties of crystal violet are given in Table 1. It is a basic, cationic, triphenylmethane dye, which is extensively used in the leather, paper, food, cosmetics, and textiles industries. It is also employed as a histological stain in the medical field and is a prime constituent of printing inks [7]. As far as toxicity of crystal violet is concerned, it is responsible for inducing mild eye discomfort [8], unpleasant light sensitivity [9] and can cause lasting damage to the cornea and conjunctiva [10]. Recent investigations reveal the carcinogenic character of crystal violet [11], while genetic alterations [12], effects on mucous membranes, and possible allergies [13] are other complications to living beings on ingestion or contact with crystal violet. Hence, the presence of crystal violet in aquatic environments poses considerable ecological and health risks to aquatic life and its undeniable presence in the human food chain is also a serious concern. Thus, its elimination from water and wastewater is a necessity, which should be tackled by developing a safe, economical, and versatile water treatment method.

Several approaches—including electrochemical treatment, ozonation, coagulation, membrane filtration, catalytic degradation, precipitation, ion exchange, and adsorption—have been employed for the eradication of various organic dyes from water and wastewater. However, the majority of these procedures are costly and demand large amounts of energy [14]. It is also pertinent to note that the large size of dye molecules complicates the selection of a safe water treatment procedure as many of these processes fragment the bulky dye compound into smaller more toxic molecules. To address these critical concerns, adsorption is considered a safer and more reliable method, and because of this, it has gained international interest. Possessing some special qualities, such as simplicity of use, low cost, absence of hazardous by-products, as-is recovery of the dye molecule, and versatility of the process, adsorption is recognised as one of the most successful physicochemical techniques in the treatment of dye-polluted water [15]. The approach is especially effective for dye adsorption since the molecule may be carefully recovered without fragmentation or altering its composition. As a result, the risk of secondary pollution is reduced, and the expensive dye itself can be recovered [16].

Adsorption is the accumulation of adsorbate particles on the surface of an adsorbent. In an adsorption process selection of an adsorbent is always a challenge as it decides the economics of the overall process. Various agricultural, industrial, and naturally-occurring waste materials have been used as adsorbents for water treatment [17,18,19,20,21,22,23,24,25,26,27,28,29]. However, there is still a need to develop new adsorbents which are cost-effective, have high extraction capacity, and are robust so that they can be reused for several cycles. Through this research work, we establish that ordered mesoporous carbon (OMC) is a very suitable adsorbent choice for water treatment because of its uniformity, high surface area, large pore volume, nanoscale pore structure, thermal and mechanical stability, and surface chemistry [30,31,32].

The focus of our research is to determine the ability of OMC to eliminate the dye, crystal violet, from aqueous solution through adsorption and also to determine various adsorption, kinetics, and thermodynamic parameters for the uptake of crystal violet by OMC. A survey of the literature reveals that removal of this dye from water or wastewater has never been attempted by using any type of OMC as adsorbent. Interestingly, the OMC developed in the present study does not suffer from any metal or halide contamination.

## 2. Results

### 2.1. Characterisation of the Metal and Halide Free OMC

To confirm the structure and composition of the OMC material, it was characterised by Fourier-transform infrared (FT-IR) spectroscopy, X-ray diffraction (XRD), scanning and transmission electron microscopy (S/TEM), the latter coupled with energy-dispersive spectroscopy (EDS), and nitrogen gas physisorption.

#### 2.1.1. Fourier-Transform Infrared Spectroscopy and X-ray Diffraction

**Figure 2:**
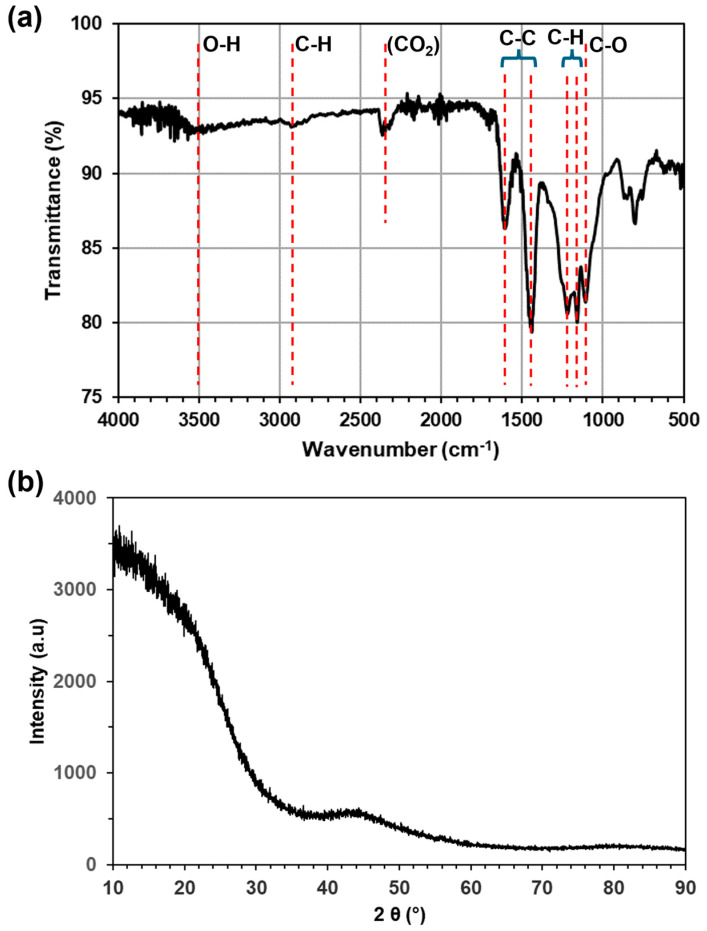
(**a**) FT-IR spectrum and (**b**) XRD pattern of the OMC material.

The FT-IR spectrum of the as-prepared OMC material is presented in Figure 2a. As indicated on the figure, the bands between 1100 and 1300 cm^−1^ are assigned to in-plane bending of C–H groups while the resonances at 1445 and 1609 cm^−1^ correspond to the C–C stretching in the aromatic rings. Bands around 1100 cm^−1^ and 2870 cm^−1^ relate to the presence of C–O and C–H stretches respectively, in the phenol, while the weak, broad band around 3500 cm^−1^ confirms the presence of the –OH group (the two peaks around 2450 cm^−1^ are due to atmospheric CO_2_ and can be disregarded). IR spectroscopy thus clearly indicates the presence of aromatic rings and of C–OH, C–H and ether bonds in the pore walls of the OMC material.

Figure 2b presents the XRD pattern of the as-prepared OMC material. The lack of sharp features and the very broad peaks at around 2θ = 10 and 44° are indicative of the essentially non-crystalline, amorphous nature of the OMC, consistent with a carbon-based material with very little long-range order.

#### 2.1.2. Electron Microscopy

**Figure 3:**
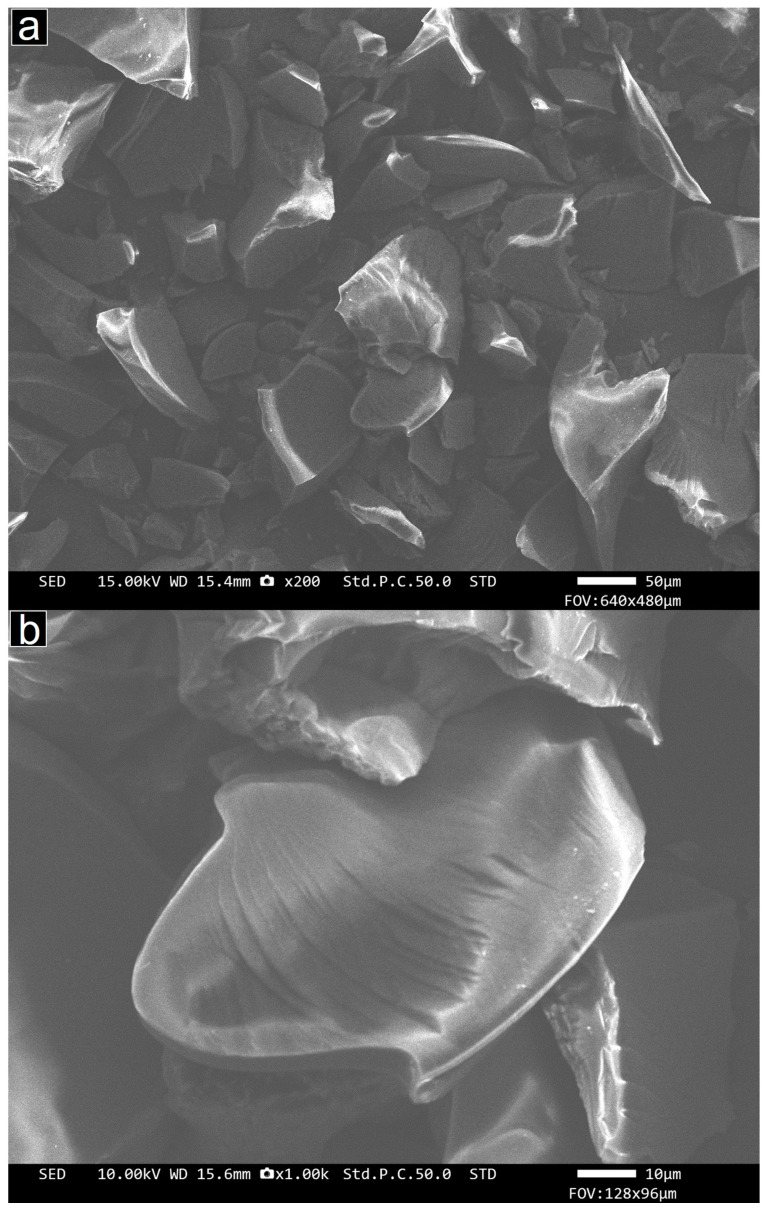
SEM images of the OMC material taken in secondary electron mode at (**a**) low and (**b**) intermediate magnification.

**Figure 4:**
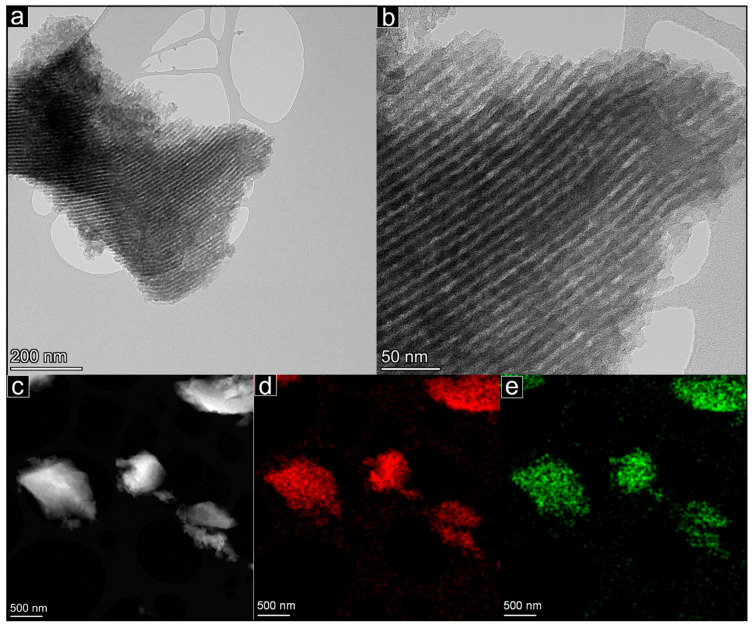
OMC material imaged in: (**a**,**b**) TEM mode, showing long, cylindrical parallel pores in profile view; (**c**) STEM mode, with corresponding EDS elemental maps showing the spatial distribution of (**d**) C; and (**e**) O.

The general form of the OMC material is seen in Figure 3, as recorded in the SEM instrument. These images show the typical chunk-like morphology of the particles, which have sizes of several tens of micrometers. Intermediate- and high-magnification images of the OMC materials recorded in TEM mode are presented in Figure 4a,b. These show that the particles consist entirely of the typical long, parallel cylindrical pores (light contrast) of the OMC and the pore walls (dark) between them, seen here in profile (side-on) view. These images confirm the expected long-range order of the uniform, parallel mesopores, which are seen to have diameters of a few nanometres. Figure 4c–e present a lower magnification image including several OMC particles taken in STEM mode and corresponding elemental maps showing the distribution of the C and O across this region of interest. These elements are spatially coincident and match the image intensities seen in the electron image in Figure 4c, indicating that the material has a homogenous chemical composition. This is quantified as around 82% C and 18% O by mass. The presence of O is consistent with the reagents used in the preparation method and with the detection of –OH and C–O–C groups by FTIR.

#### 2.1.3. Nitrogen Gas Physisorption

**Figure 5:**
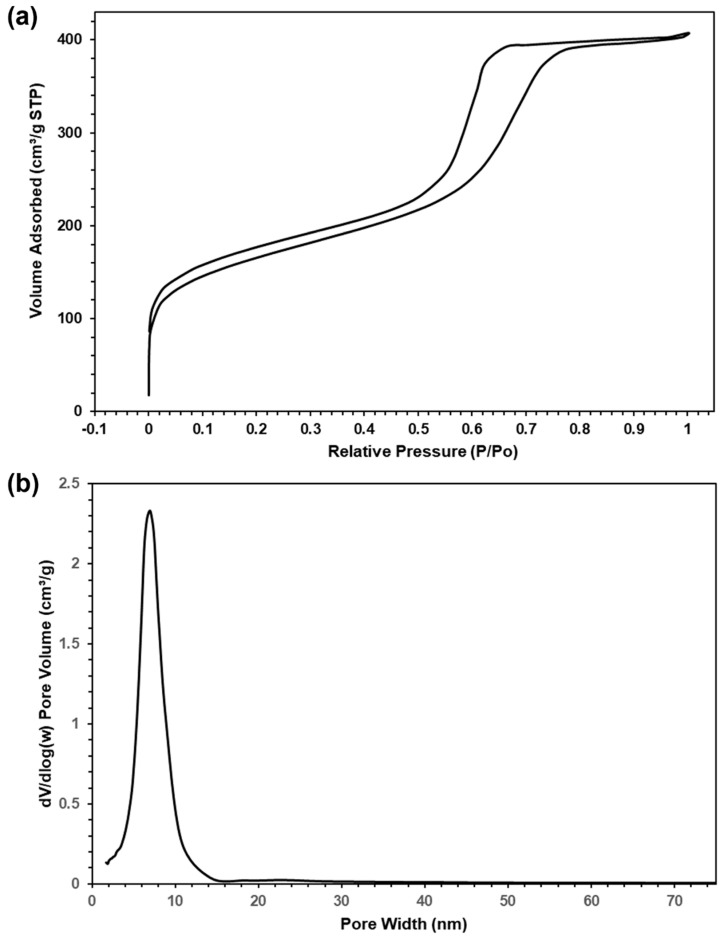
(**a**) Nitrogen physisorption isotherm of OMC and (**b**) corresponding plot of pore size distribution.

The adsorption and desorption isotherms for N_2_ physisorption are given in Figure 5, along with the corresponding pore size distribution plot obtained from the adsorption branch. The form of the isotherms indicates Type IV behaviour and H1-type hysteresis with a relatively sharp capillary condensation step at relative pressure, 0.4–0.8. This confirms that the material contains mesopores of cylindrical shape. BET analysis of these data provides values of specific surface area and specific pore volume of 586 m^2^g^−1^ and 0.585 cm^3^g^−1^, respectively, which are typical of these OMC materials. The pore size distribution presented in Figure 5b shows a single intense, sharp peak, attesting to the uniform dimensions of the mesopores and the absence of other pore structures in the material. This peak is centred at 7.0 nm, which again is typical of this class of OMC materials and is consistent with the mesopore structure seen in the electron microscopy images.

#### 2.1.4. Interpretation of Characterization Results

On the basis of the analysis of the OMC material, it is clear that it is a fine powder whose particles are mesoporous—possessing an ordered array of long cylindrical pores of ~7 nm diameter—and that the pore walls contain mainly carbon but with significant oxygen content, which is held in the C–OH, C–H and ether groups detected in FTIR. None of the techniques indicate the presence of metal or halide species in this material, which are often present in OMCs prepared by conventional methods, which typically use HCl and/or NaOH catalysts [33].

### 2.2. Batch Adsorption Studies

The batch adsorption of the Crystal Violet dye over the metal- and halide-free OMC is examined in three segments. Firstly, preliminary investigations were carried out to optimize various parameters which affect the adsorption. Based on the optimum values of these parameters, the dye–OMC adsorption behaviour was tested against different adsorption isotherm models and various thermodynamic parameters were calculated. The third part of the study includes measurements of kinetics, diffusion and mechanism of the adsorption process.

#### 2.2.1. Preliminary Tests under Batch Conditions

For an exhaustive adsorption analysis, it is essential to first examine and optimize the effect of various physico-chemical parameters, which can strongly affect the accumulation of adsorbate over the adsorbent surface. Variables chosen for the study are pH of the solution, adsorbent dosage, dye concentration and contact time.

##### Effect of Solution pH

**Figure 6:**
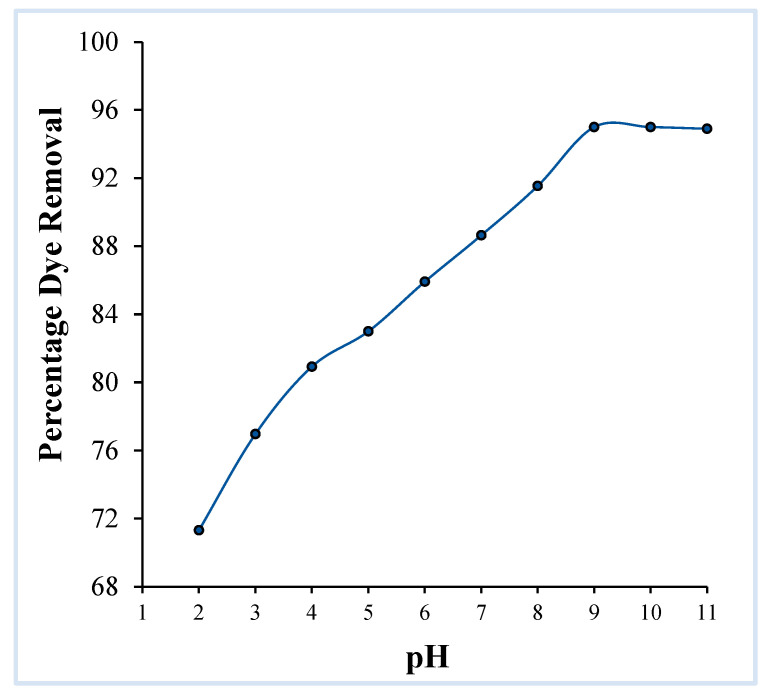
Effect of pH on the adsorption of the crystal violet dye over OMC (adsorbent dosage: 0.025 g/20 mL, initial dye concentration: 4 × 10^−5^ M, contact time: 2 h).

The pH of the dye solution has a crucial effect on the adsorption system. Using 0.1 N NaOH and 0.1 N HCl, the pH values of dye solutions were altered from 2 to 11 and dye uptake on the OMC was examined for each pH value. It was discovered that as the pH is increased from 2 to 9, dye adsorption increases, reaching a maximum sequestration of crystal violet of 950 mg/g at pH 9 (Figure 6). Beyond pH 9, adsorptive removal remains constant.

**Figure 7:**
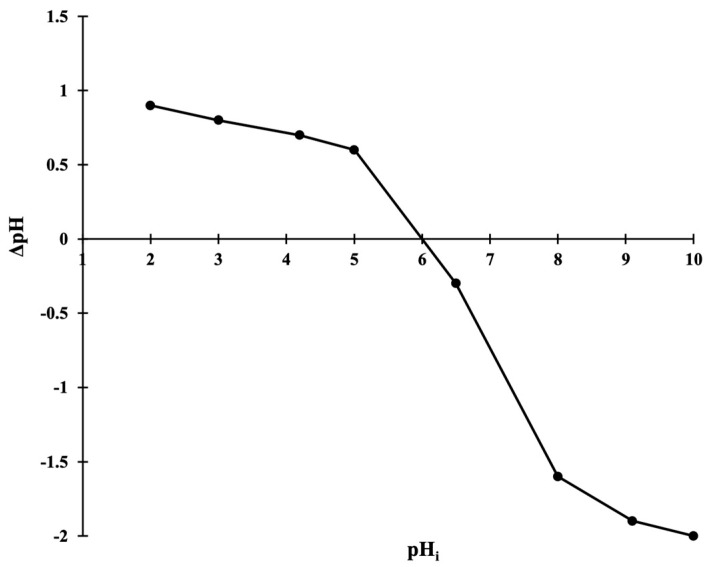
Determination of pH_(ZPC)_ of OMC.

To determine the point of zero charge of the adsorbent (pH_PZC_) a plot of change in pH (ΔpH) versus initial pH of the solution (pH_i_) was constructed (Figure 7). This clearly shows that an intersection on the *x*-axis, which corresponds to a net zero charge on the adsorbent (pH_PZC_), is obtained at pH 6.0. Thus, below pH 6.0, when pH < pH_PZC_, the surface charge of the OMC becomes positive, while above pH 6.0, when pH > pH_PZC_ the material bears negative charge.

On the basis of these results, it can be interpreted that the lesser adsorption of the crystal violet in strong acidic medium is due to the basic (cationic) nature of the dye and the fact that presence of similar charges on both the adsorbate and the adsorbent is not conducive to accumulation of crystal violet over the OMC surface. As the pH of the solution increases, OMC develops more and more negative charge, and as a result the dye–adsorbent interaction increases, which is observed as higher dye uptake. The uptake becomes more significant beyond pH 6.0, i.e., at the pH_PZC_ of the OMC. In the adsorption process, the pH of the medium plays a very prominent role because it determines the ionization state of the adsorbate molecule. Thus, in the case of adsorption of dyes, the pKa of the dye is more significant and adsorption does not depend only upon the pH_zpc_ of the adsorbent. Crystal violet exhibits a pKa of 8.64, therefore at pH 9 the greatest adsorption of the dye is observed and beyond this point a plateau is attained which can be attributed to saturation of accessible sites on the OMC. In the light of these findings, dye solution of pH 9 was used for all further studies.

##### Effect of Adsorbent Dosage

**Figure 8:**
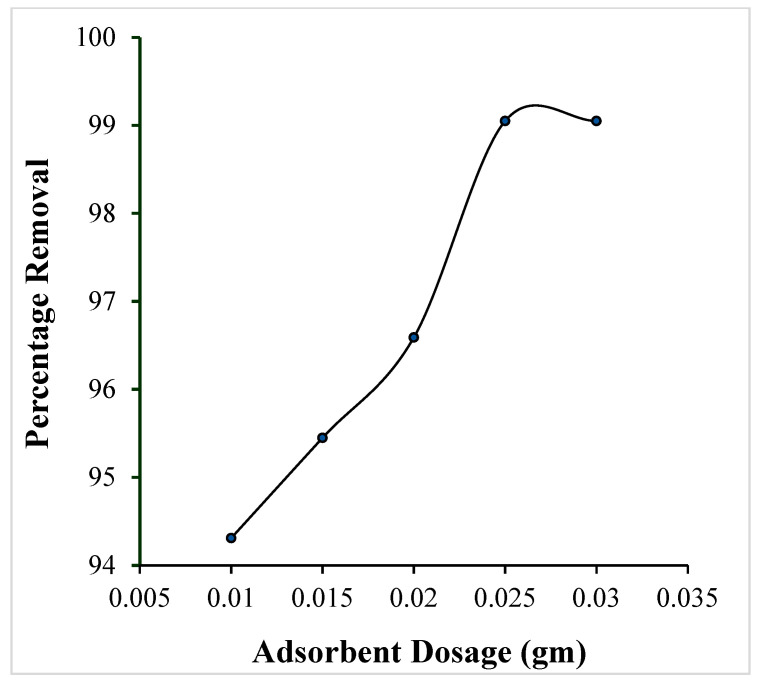
Effect of adsorbent dosage on the adsorption of the crystal violet dye over OMC (pH: 9, initial dye concentration: 4 × 10^−5^ M, contact time: 2 h).

To investigate the effect of the amount of adsorbent on the adsorption process, dye uptake was recorded while varying the amount of OMC from 0.01 to 0.03 g. Figure 8 shows that over the dosage range from 0.01 g to 0.025 g a linear increase in the percentage removal of the dye is observed. The increase in accumulation of dye is due to a rise in the number of adsorption binding sites as the proportion of OMC increases. Beyond 0.025 g, saturation is observed which may be attributed to the exhaustion of adsorbent material resulting in non-availability of adsorption sites on the OMC.

##### Effect of Dye Concentration

**Figure 9:**
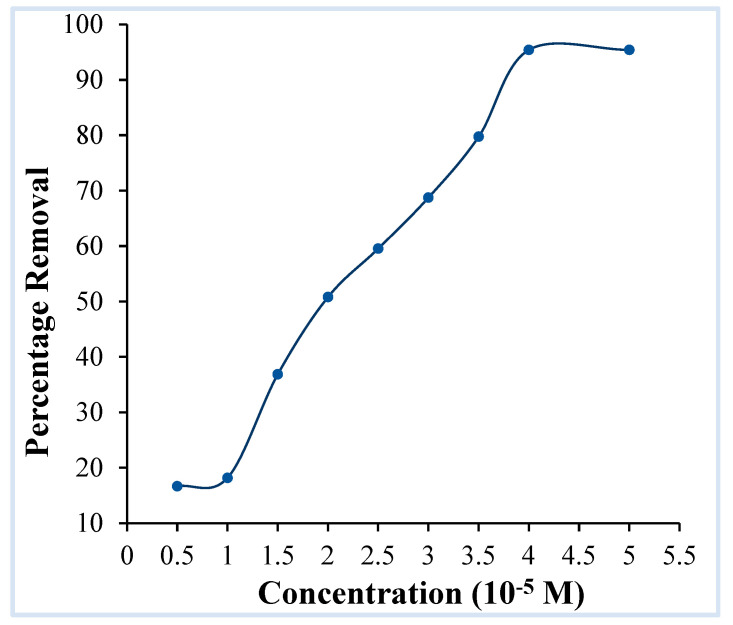
Effect of dye concentration on the adsorption of crystal violet over OMC. (pH: 9, adsorbent dosage: 0.025 g/20 mL, contact time: 2 h).

Another key component which affects the adsorption process is the dye concentration, as it creates an equilibrium in which dye molecules diffuse from the solution phase to the surface of the adsorbent. The consequence of crystal violet concentration on its accumulation on OMC was investigated across a concentration range of 0.5 × 10^−5^ M to 5 × 10^−5^ M with 0.025 g of adsorbent and at pH 9. The concentration analysis (Figure 9) demonstrates that, on increasing the concentration of the dye, the degree of adsorption increases linearly until a certain point (4 × 10^−5^ M), after which dye removal becomes steady due to saturation of adsorbent sites and as clustering of dye molecules at the solid–solution interface inhibits the accumulation of further dye molecules. In accordance with this finding, the following exploratory studies are conducted at a dye concentration of 4 × 10^−5^ M.

##### Effect of Contact Time

**Figure 10:**
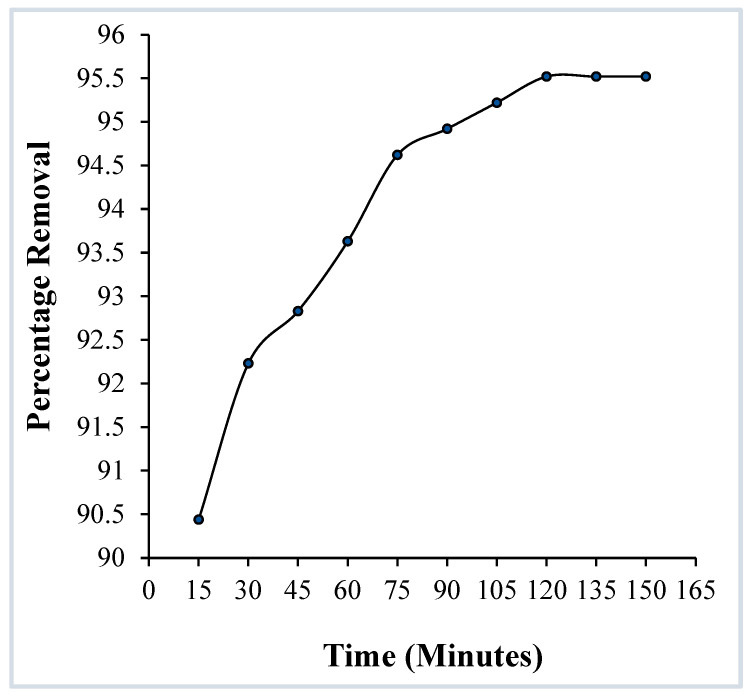
Effect of contact time on the adsorption of crystal violet over OMC. (pH: 9, initial dye concentration: 4 × 10^−5^ M, adsorbent dosage: 0.025 g).

The adsorption of crystal violet on OMC was examined as a function of contact time to establish the equilibration time for the maximum removal of the dye (Figure 10). A constant amount of adsorbent (0.025 g) in a solution with a constant pH of 9 and dye concentration of 4 × 10^−5^ M was used. Figure 10 shows that the rate of dye removal is higher initially, gradually decreases, and becomes practically constant after 120 min, indicating that the equilibrium of dye molecules between the OMC surface and the solution is attained at this time. That is, that the adsorbent has reached its saturation state.

#### 2.2.2. Isotherms Investigations

Isotherm research was conducted to determine the applicability of the reaction between adsorbate and adsorbent particles and also to understand the thermodynamics of the process. The data collected throughout the experiments under a range of isothermal conditions are used to calculate important parameters to determine whether the adsorption process is spontaneous, favourable, feasible, exothermic/endothermic, and chemical/physical nature. Langmuir, Freundlich, Temkin, and Dubinin–Radushkevich isotherm models were examined.

Adsorption is a surface phenomenon, which involves the accumulation or concentration of substances at a surface or interface. This process can occur at an interface of any two phases, such as liquid–liquid, gas–liquid, gas–solid or liquid–solid. Therefore, adsorption is a process in which the molecules or atoms of one phase interpenetrate nearly uniformly among those of another phase to form a solution with this phase. As such, the solute remaining in the solution is in dynamic equilibrium with that at the surface of the adsorbed phase. In other words, we may say that it is a process in which a certain adsorptive is selectively transferred from the fluid phase, called the adsorbate, to the surface of insoluble, rigid particles suspended in a reaction vessel or packed in a column and called the adsorbent.

Adsorption is related to the phenomenon of surface energy. In a bulk material, all the bonding requirements, whether ionic or covalent of the constituent atoms of the material are satisfied. However, atoms on the adsorbent surface experience a bond deficiency and the preferential concentration of molecules in the proximity of adsorbent surface arises. The adsorption of various substances is due to increased free surface energy of the solids because of their extensive surface. According to the second law of thermodynamics, this energy has to be reduced. This is achieved by reducing the surface tension via the capture of extrinsic substances. The exact nature of the bonding depends on the details of the species involved and is generally classified as physisorption or chemisorption.

##### Langmuir Isotherm and Thermodynamic Parameters

In 1916, Irving Langmuir developed an empirical isotherm, which depends upon the monolayer adsorption capacity of an adsorbent by assuming unimolecular layer formation of the adsorbate at adsorption saturation [34]. This sorption model is based on two hypotheses, (a) that uptake of adsorbate occurs on a homogenous surface by monolayer adsorption and (b) there is no interaction between the ions of adsorbate. The model also considers that there is no transmigration of the adsorbate in the plane of the surface of the adsorbent. The model is quite versatile, and the mechanism involved in all the adsorbate–adsorbent systems are treated as being similar.

To determine the applicability of the Langmuir isotherm, the relationship of 1/c_e_ vs. 1/q_e_ was plotted at three different temperatures. The Langmuir equation can be stated mathematically in the following way:

(1) $$\frac{1}{q_{e}}=\frac{1}{q_{o}}+\frac{1}{{\mathrm{bq}}_{o}C_{e}}$$

**Figure 11:**
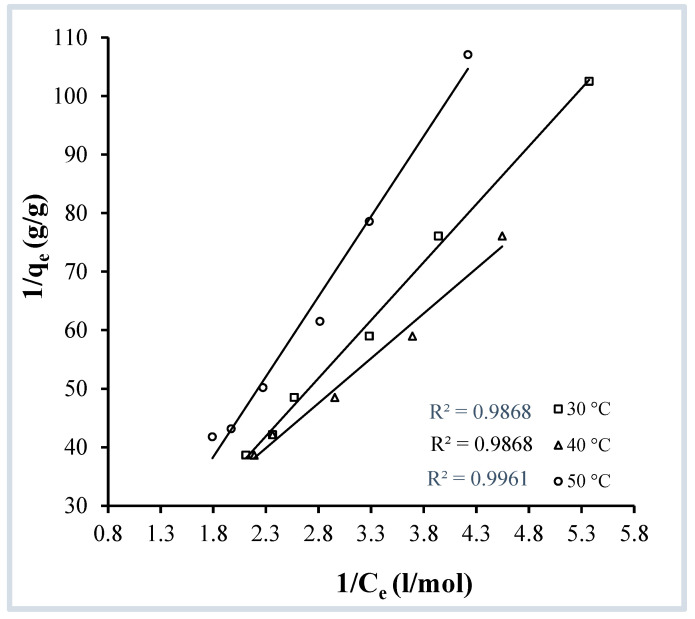
Langmuir isotherms for the adsorption of crystal violet on OMC at pH 9.

Here, q_e_ is the adsorbed quantity of adsorbate (mol·g^−1^), C_e_ is the dye’s equilibrium molar concentration (mol L^−1^), q_o_ is the maximal adsorption capacity per unit mass of the adsorbent (mol·g^−1^), the value of q_o_ is determined from the inverse of the intercept, and b is the Langmuir constant (L·mol^−1^). At each temperature (30, 40, and 50 °C), the graph of 1/c_e_ vs. 1/q_e_ (Figure 11) fits to a straight line with regression coefficient values near to unity (Table 2), demonstrating that the Langmuir isotherm is consistent with the present adsorption process. The values of the Langmuir constant, b, are determined from the intercept and slope of the straight lines and are also presented in Table 2.

To determine the favourability of the reaction, a factor known as the separation factor is calculated using the Langmuir constant (b). The separation factor (r) can be expressed as follows:

(2) $$r=\frac{1}{1+{\mathrm{bc}}_{o}}$$

For a favourable reaction, the separation factor should lie between 0 and 1. Considering the values of the separation factor given in Table 2, the adsorption of crystal violet over OMC is favourable at all three temperatures used (30, 40, 50 °C).

The thermodynamic parameters, changes in entropy (ΔS°), enthalpy (ΔH°), and Gibbs free energy (ΔG°) were derived using the Langmuir constant (b) in the following well-known expressions [35]:

(3) $$∆H{}^{\circ}=-R\left(\frac{T_{2}T_{1}}{T_{2}-T_{1}}\right)\times \ln\left(\frac{b_{2}}{b_{1}}\right)$$

(4) ∆G°=−RTlnb

(5) $$∆S{}^{\circ}=\frac{\left(∆H{}^{\circ}-∆G{}^{\circ}\right)}{T}$$

In these three reactions, R is the universal gas constant (J·mol^−1^ K^−1^). T is the temperature in K, and b is the Langmuir constant (L·mol^−1^). Positive and negative values of ΔH°, ΔS°, and ΔG° reflect the probability that the reaction is endothermic or exothermic, whether there are increases or decreases in the system’s disorder, and whether the reaction is spontaneous or not, respectively. At all three temperatures used, negative values of ΔG° indicate the spontaneity of the process, positive values of ΔH° imply its endothermic nature, while positive values of ΔS° confirm the increased of disorder during the adsorption process (Table 3).

##### Freundlich Isotherm

In the Freundlich model [36], it is considered that the binding affinities on the adsorbent surface vary with the interactions between the adsorbed molecules. Consequently, the sites with stronger affinity are occupied first. The equation describing the Freundlich model represents adsorption of solutes from a liquid to a solid surface. This experimental model can be applied to non-ideal adsorption on heterogeneous surfaces as well as multilayer adsorption. The Freundlich model is chosen to estimate the extent of adsorption of the adsorbate on the adsorbent surface.

The Freundlich isotherm model [36] is based on the assumption that adsorbate molecule accumulation occurs on a heterogeneous surface by multilayer adsorption. The linear form of the well-established Freundlich equation can be expressed as

(6) $${\mathrm{logq}}_{e}=\log k_{f}+\left(\frac{1}{n}\right){\;\mathrm{logC}}_{e}$$

**Figure 12:**
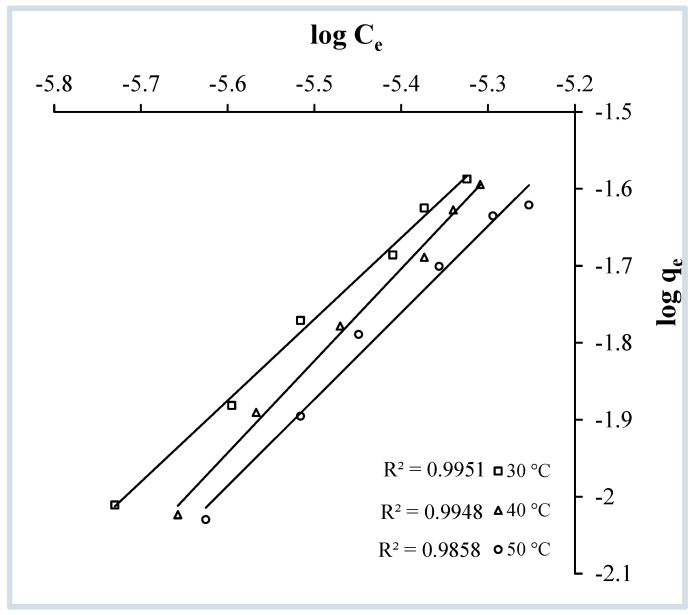
Freundlich isotherms for the adsorption of crystal violet over OMC at pH 9.

Here, The terms q_e_ and C_e_ have the same significance as in Equation (1). k_f_ and n are the maximum adsorption capacity and Freundlich constant, respectively. To verify the Freundlich adsorption isotherm model, log C_e_ vs. log q_e_ is plotted (Figure 12). At each temperature, the value of k_f_ is determined from the intercept, and n is determined from the slope of the plot. The Freundlich Isotherm plots also give straight lines with the coefficients of regression very close to one (Table 2), which means that this isotherm model is considered to be valid and implies that the surface of the adsorbent is heterogeneous.

##### Temkin Isotherm

In 1940, Temkin and Pyzhev [37] investigated the impact of several indirect adsorbate/adsorbate interactions on the adsorption isotherm and proposed that the heat generated during adsorption is associated with every molecule accumulating on the adsorbent surface and that, as a result of intermolecular interactions, the heat would drop linearly with increasing coverage. Moreover, the adsorption process is characterized by a uniform distribution of the binding energies, up to maximum binding energy. The Temkin isotherm model has the following linear form:

(7) $$q_{e}=k_{1}{\mathrm{lnk}}_{2}+k_{1}{\mathrm{lnC}}_{e}$$

**Figure 13:**
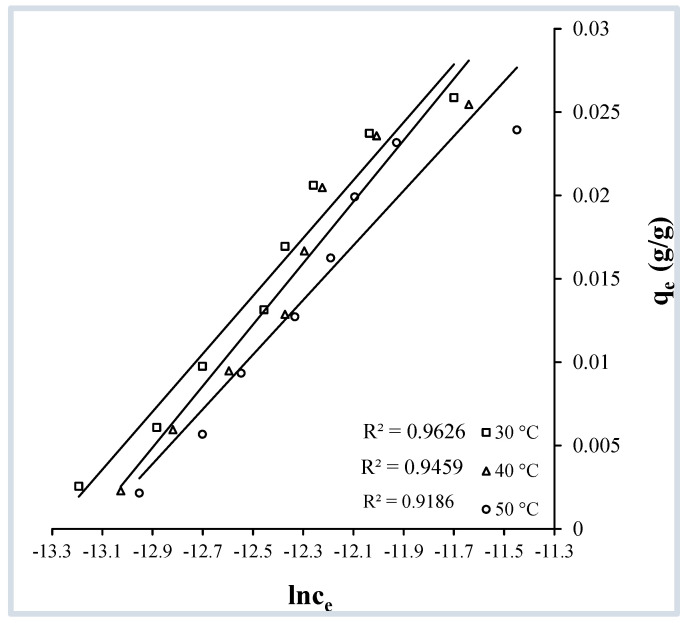
Temkin isotherms for the adsorption of crystal violet over OMC at pH 9.

**Figure 14:**
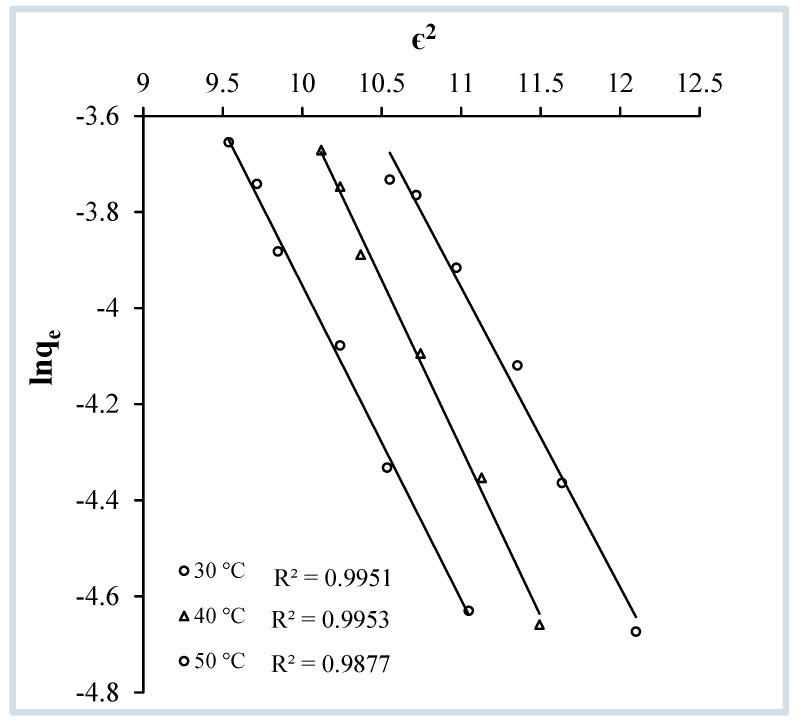
Dubinin–Radushkevitch isotherms for the adsorption of crystal violet over OMC at pH 9.

Notations q_e_ and C_e_ in this equation possess the same meaning as above, while k_1_ and k_2_ are Temkin coefficients (L·mol^−1^). A plot of lnC_e_ vs. q_e_ is constructed (Figure 13) and linear fits, with reasonably acceptable regression coefficients, are obtained at all three temperatures (30, 40, and 50 °C) demonstrating the applicability of Equation (7), and of the Temkin model in the present case. This reveals that the energy dispersion is equivalent between the adsorbent and adsorbate associations. Values of k_1_ and k_2_ derived from the intercept and slope of the linear fits of Figure 14 are presented in Table 2.

##### Dubinin–Radushkevitch (D-R) Isotherm

The D-R isotherm model has been designed to account for the effect of the pore size distribution in adsorbents and considers the distribution of pores in adsorbents to obey a Gaussian energy distribution [38]. The isotherm also provides valuable insight to calculate mean sorption energy “E”, which in turn diagnoses the nature of the interaction between the adsorbate and adsorbent and determines whether the process is chemisorption or physisorption.

The D-R isotherm equation can be expressed quantitatively as follows:

(8) $${\mathrm{lnq}}_{e}=\ln X_{m}-\beta \varepsilon^{2}$$

In this equation, X_m_ is the maximal sorption capacity (mol·g^−1^), β is the activity coefficient (mol^2^ J^−2^), and q_e_ has the same meaning as in Equation (1) (in mol·g^−1^). β is determined from the slope and X_m_ is found from the intercept of a plot of ɛ^2^ versus lnq_e_. At all three temperatures the ɛ^2^-versus-lnq_e_ plot exhibited straight lines with regression coefficient values close to unity, verifying the applicability of DR adsorption isotherm in the present case (Figure 14). In this expression (Equation (8)) ɛ is the Polanyi potential, which can be found using the following equation:

(9) $$\varepsilon =\mathrm{RTln}\left(1+\frac{1}{C_{e}}\right)$$

In Equation (9), R and T are the universal gas constant (J·mol^−1^ K^−1^) and temperature (K), respectively. C_e_ is the concentration at equilibrium (mol·L^−1^). The work required to remove a molecule to infinite distance from its site in the sorption region, regardless of temperature, is known as the Polanyi sorption potential (ɛ) [39]. The isotherm’s usefulness varies depending on the particular type of adsorption process, with mean sorption energy (E) is the key determinant in highlighting whether chemical or physical adsorption is occurring. The form is as follows:

(10) $$E=\frac{1}{\sqrt{-2\beta }}$$

In the process of adsorption, physisorption is dominant when the mean sorption energy “E” is less than 8 kJ/mol, while chemisorption is dominant when “E” is 8 to 16 kJ/mol. The obtained values of E are greater than 8 kJ/mol at temperatures 30, 40, and 50 °C (Table 2) proving that the crystal violet molecules adhere to the OMC through chemical bonding and the adsorption process can be safely interpreted as chemisorption.

#### 2.2.3. Adsorption Kinetics

The kinetics of the crystal violet–OMC system was investigated to assess the order of the reaction and rate constant of the rate-controlling step of the reaction. To obtain the kinetic data, contact time experiments were performed at three different temperatures, 30, 40, and 50 °C. The acquired data were then evaluated through Lagergren’s pseudo-first-order [40,41], Ho–McKay pseudo-second-order [42,43], and Weber and Morris intraparticle diffusion models [44,45]. The linear representations of these models are given in the following equations [40,41,42,43,44,45]:

(11) $$\frac{t}{q_{t}}=\frac{1}{k_{2}q_{e}^{2}}+\frac{t}{q_{e}}$$

(12) $$\log\left(q_{e}-q_{t}\right)={\mathrm{logq}}_{e}-\frac{k_{1}}{2.303}\times t$$

(13) $$q_{t}=K_{\mathrm{id}}t^{0.5}+C_{i}$$

In the above equations, q_e_ and q_t_ are the adsorption capacities at equilibrium and time “t”, respectively; k_1_ (min^−1^) and k_2_ (g·mol^−1^ min^−1^) are the pseudo-first-order rate constant and pseudo-second-order rate constant, respectively. K_id_ (mol·g^−1^·min^−1/2^) denotes the constant of intra-particle diffusion, and C_i_ denotes the constant and its value is equal to the thickness of the boundary layer developed at the interface of dye solution and the OMC.

**Figure 15:**
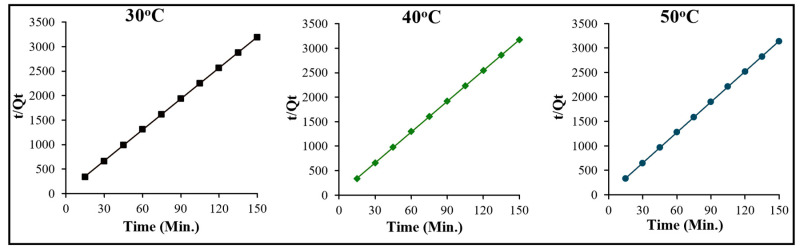
Pseudo-second-order plots for the adsorption of crystal violet over OMC at the temperatures indicated.

**Figure 16:**
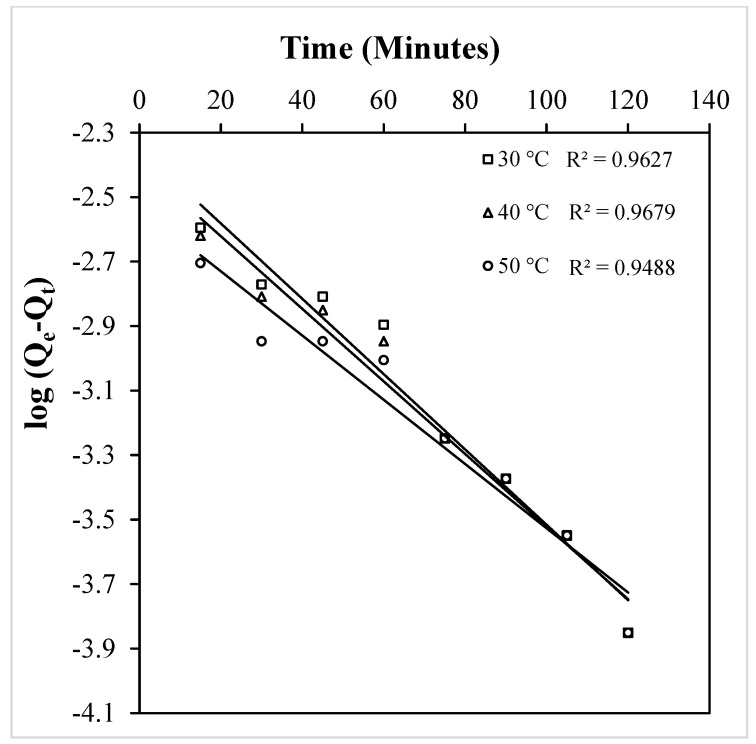
Pseudo-first-order plot for the adsorption of crystal violet over OMC at the temperatures indicated.

Plots of time (t) versus t/q_t,_ yield straight lines with regression coefficient values of unity at all the three temperatures (Figure 15), demonstrating the applicability of the pseudo-second-order rate expression in this situation. The k_2_ values derived from the slope and intercept of Figure 15 at 30, 40, and 50 °C are found to be 12.93, 14.08, and 17.28 min^−1^, respectively. Attempts were also made to test for pseudo-first order kinetics and the time versus $\log\left(q_{e}-q_{t}\right)$ graphs were plotted at each temperature. Figure 16 clearly shows that none of the straight lines possess satisfactory regression coefficient values, and therefore, the kinetics of the crystal violet–OMC adsorption is interpreted as definitively pseudo-second–order.

**Figure 17:**
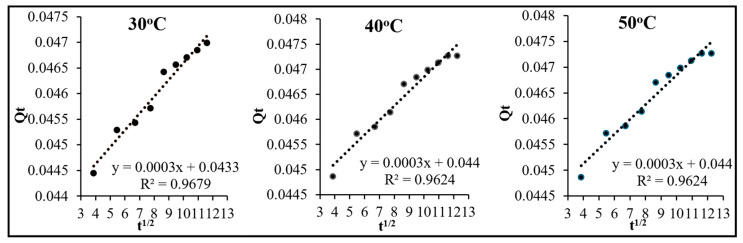
Intra-particle diffusion plots for the adsorption of crystal violet over OMC at the temperatures indicated.

Making use of Equation (13), an intra-particle diffusion plot of t^1/2^ versus q_t_ (), was made (Figure 17) to clarify the type of diffusion occurring in the process, that is whether adsorption proceeds via intra-particle, film, or particle diffusion. The Weber and Morris model [44,45] explains that a value of zero for the intercept (C_i_) of this plot would confirm involvement of intra-particle diffusion, while a non-zero value would indicate either film or particle diffusion. In the present case, at all the temperatures, the intercept of the straight line fits presented in Figure 17 are non-zero, indicating that intra-particle diffusion is not the rate-determining step in this reaction and we have to further investigate whether the adsorption of crystal violet over OMC is governed by film diffusion or particle diffusion on the basis of models suggested by Boyd et al. [46] and Reichenberg [47].

##### Reaction Mechanism Explication

It is well known that the adsorption of an organic or inorganic species at the surface of a porous adsorbent normally involves three steps [48]: (a) film diffusion, involving transport of adsorbate to the external surface of the adsorbent; (b) particle diffusion, mainly involving transport of adsorbate within the pores of the adsorbent; and (c) adsorption on the adsorbent’s interior surface. The process (c) is considered very fast and cannot be treated as the rate limiting step [48]. In the steps (a) and (b) it is likely that transportation of some of the adsorbate can be to the internal surface, while major adsorption takes place at the external surface of the adsorbent, or vice versa. Thus, there are the following three possibilities:

I. External Transport < Internal Transport (film diffusion is rate limiting);
II. External Transport > Internal Transport (particle diffusion is rate limiting);
III. External Transport ≈ Internal Transport, where adsorption is not feasible at a significant rate due to the formation of a liquid film over the sorbent particles.

To discover the mechanism involved in the ongoing adsorption the kinetic data are further fitted to the Reichenberg and Boyd model [46], which is based on following mathematical expressions:

(14) $$F=\frac{q_{t}}{q_{\infty }}$$

(15) $$F=1-\frac{6}{\pi^{2}}\sum_{\infty }^{1}\left(\frac{1}{n^{2}}\right)e^{\left(-n^{2}B_{t}\right)}$$

(16) $$B_{t}=\frac{\pi^{2}D_{i}}{\left(r_{0}^{2}\right)}=\mathrm{Time}\;\mathrm{constant}$$

In these Equations (14)–(16), q_t_ and q_∞_ represent the adsorption capabilities of the adsorbent at time “t” and “infinity”, respectively, D_i_ (cm^2^s^−1^) is the effective diffusion coefficient within the adsorbent pores, while n denotes the Freundlich constant, F is the degree of achievement of fractional equilibrium at time “t”, and B_t_ is the time constant. Values of B_t_ are dependent upon F and are derived from Reichenberg’s table [47].

**Figure 18:**
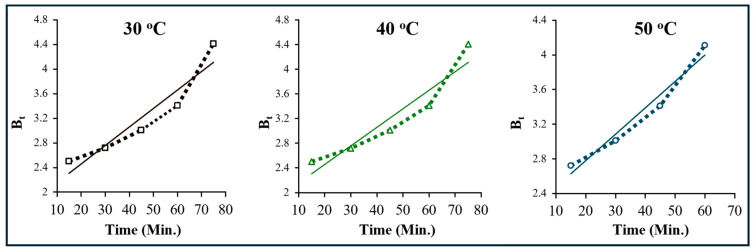
Time-versus-B_t_ plot for crystal violet adsorption over OMC at the temperatures indicated.

The graph of time (t) versus B_t_ can be used to distinguish between film and particle diffusion steps of the adsorption process. The straight line fits obtained from the graph, when passing through the origin, mean particle diffusion is the operative mechanism. However, if straight lines are not obtained or the straight lines do not pass through the origin, film diffusion is operative. Figure 18 clearly reveals that the adsorption of crystal violet on OMC obeys film diffusion at all the temperatures and external transport of the dye molecules dominates over internal transport.

#### 2.2.4. Comparative Batch Adsorption

The adsorption capability of OMC for the dye, crystal violet, has been compared to that of other adsorbents studied in the literature [49,50,51,52,53,54,55,56,57,58,59,60,61] and is presented in Table 4. It is clear from the data that crystal violet exhibits maximum adsorption capacity towards OMC as compared to other natural and synthetic adsorbents. It is also pertinent to note that despite different optimum physico-chemical parameter values, in all the cases, pseudo-second-order kinetics are followed and each adsorption process is spontaneous, endothermic, and observes increasing disorder.

### 2.3. Continuous Adsorption/Desorption in a Fixed Bed Column

#### 2.3.1. Dye Adsorption in OMC Fixed Bed Column

In the present work, a mass transfer kinetic approach as postulated by Weber [61] has been applied and parameters for the bulk dye removal were evaluated. The design and the operation of Weber’s approach results in a breakthrough curve, which allows determination of various important column parameters including its percentage saturation. The breakthrough curve is the plot of the volume of the exiting dye solution versus its concentration. Its S-shape is influenced by the individual transport processes in the column and by the nature of the adsorbent [62,63].

**Figure 19:**
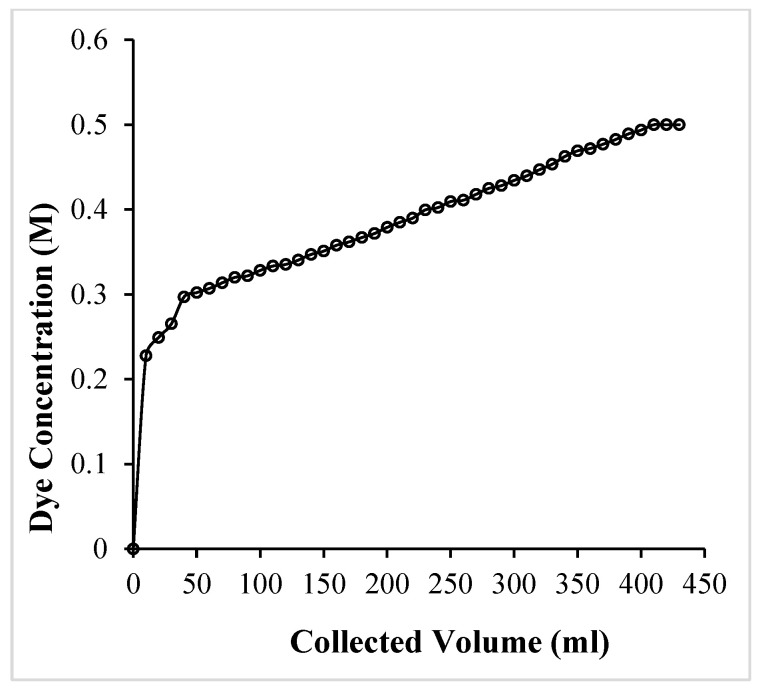
Breakthrough curve for the adsorption of crystal violet in an OMC column.

Figure 19 presents the breakthrough curve obtained during adsorption of crystal violet over a fixed bed of OMC. The breakthrough point is considered to be the value when effluent concentration (C_x_) closely approaches the initial dye concentration (C_o_), and this is the stage of complete exhaustion of the adsorbent. The segment of the curve between C_x_ and C_b_ (concentration of the dye at breakthrough point) defines the formation of the primary adsorption zone and is assumed to have a constant length. The total time involved in the formation of the primary adsorption zone (t_x_) is V_x_/F_m_, where V_x_ is the volume (ml) to reach concentration C_x_, and F_m_ is the mass flow rate (mg·cm^−2^·min^−1^). In the present case, V_x_ is 410 mL and F_m_ is calculated as 1.167 mg·cm^−2^·min^−1^ (Table 5).

The time required for the primary adsorption zone to move down its length (t_δ_) in the column is given as [61,62,63]

(17) $$t_{\delta }=\frac{V_{x}-{\;V}_{b}\;}{F_{m}}$$

where V_b_ is the volume (mL) of the dye required to reach the breakthrough point (40 mL). Thus, for a length D’ of the adsorbent bed (0.9 cm), the following mathematical expressions are applied:

(18) $$\frac{\delta }{D}=\frac{t_{\delta }\;}{t_{x}-t_{f}}=\frac{t_{\delta }\;}{t_{x}+t_{\delta \;}\left(f\;-1\right)}=\frac{\left(V_{x}-{\;V}_{b}\right)}{V_{b}+\;f\;\left(V_{x}-{\;V}_{b}\right)}$$

where t_f_ is time required to form the primary adsorption zone at the adsorbent, “f” is the fractional capacity of the adsorbent column at the breakpoint (Table 6) and is defined as:

(19) $$f=1-\frac{t_{f}\;}{t_{\delta }\;}=\frac{m_{s}\;}{\left(V_{x}-{\;V}_{b}\right)\;C_{o}}$$

where m_s_ is the amount of the dye adsorbed in the primary adsorption zone and C is the concentration of the dye loaded to the column. The fractional capacity (f) is used to calculate the percentage saturation of the adsorbent in the fixed bed column and was found to be around 97% (Table 6) by the following mathematical expression:

(20) $$\mathrm{Percentage}\;\mathrm{Saturation}=\frac{D+\;\delta \;\left(f\;-1\right)\;}{D}\times 100$$

#### 2.3.2. Regeneration of Column and Dye Recovery

To develop an economically viable process for industry, recovery of the costly dye material by desorbing from the adsorbent bed is an important step [62,63]. After complete desorption of the dye, the fixed bed of adsorbent is regenerated and several adsorption/desorption cycles can be performed on the same column [62,63]. This also helps in establishing the robust nature of the adsorbent.

**Figure 20:**
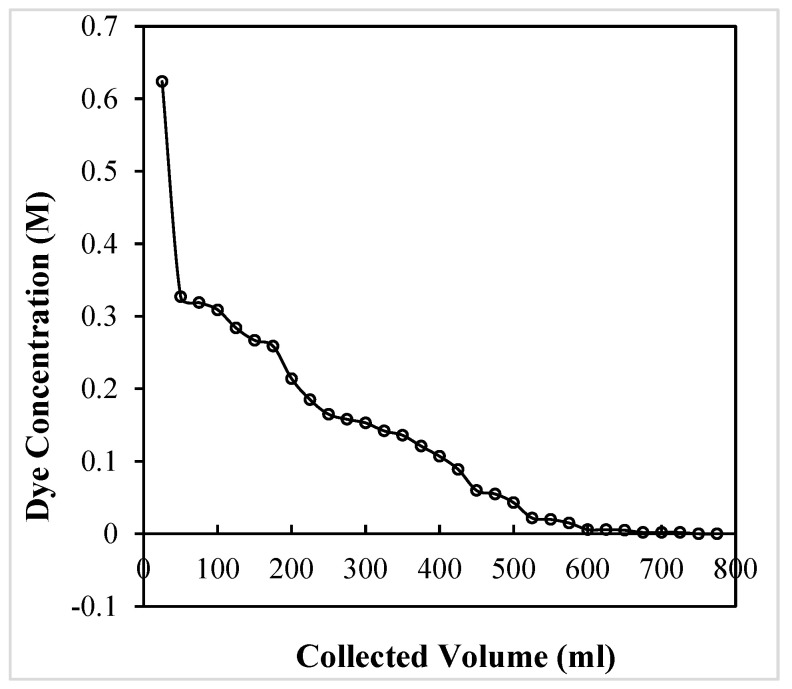
Desorption of crystal violet from exhausted OMC column.

Once the adsorption process is completed and the OMC column is fully exhausted, dil. H_2_SO_4_ is passed through the column to desorb the dye. During the adsorption process, a total of 53.343 mg of dye was adsorbed by the OMC, and of this, 12.314 mg (23%) was obtained in the first 50 mL aliquot, while around 50% of the dye recovery could be made in 150 mL of the collected effluent (Figure 20). Figure 20 also shows that almost 100% dye recovery (53.050 mg, 99.47%) was achieved in the first desorption cycle where a total of 725 mL of the effluent volume was collected.

#### 2.3.3. Determination of Column Efficiency

**Figure 21:**
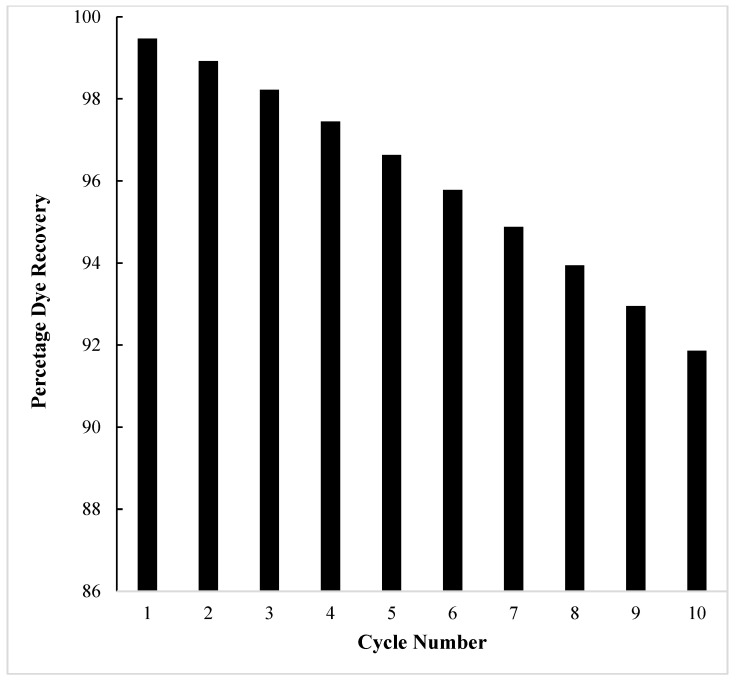
Column efficiency as a function of number of adsorption–desorption cycles.

By carrying out several adsorption–desorption cycles on the fixed-bed column, its efficiency can be determined. In the present case, a total of 10 cycles were examined, and on the basis of results obtained (Figure 21)—where almost 92% of dye was recovered—it can be safely interpreted that OMC is a very robust material, which can very efficiently work for several adsorption–desorption cycles in industrial applications.

## 3. Materials and Methods

### 3.1. Chemicals

All of the chemicals employed in the synthesis of the OMC, ammonium hydroxide, formaldehyde, resorcinol, oxalic acid and Pluronic F127, were procured from M/s Sigma-Aldrich (St. Louis, MO, USA). The dye crystal violet, dilute HCl and dilute NaOH were purchased from M/s Merck, Bengaluru, India. All the chemicals were utilised without being purified further. Double-distilled water used in all the studies was prepared in the laboratory.

### 3.2. Synthesis of Metal and Halide Free OMC

The synthesis of OMC follows the method developed by Sakina and Baker [64]. This method is notable in its easy applicability and in the fact that it avoids the use of metal- and halide-containing catalysts and therefore results in OMC products free from these contaminants. In this method, a non-ionic copolymer surfactant—Pluronic F127—is used to construct a soft template. Within the range of concentration used, the F127 copolymer forms cylindrical micelles which are allowed to self-assemble into a hexagonally packed structure. The condensation polymerisation reaction of resorcinol and formaldehyde is carried out in the presence of this self-assembled array forming polymer material between the micelles. Ammonium hydroxide and oxalic acid are used to catalyse the polymerisation. Both the catalysts are completely degradable, unlike the mineral acids and bases used in conventional methods. Heating steps are then applied to remove the surfactant and further condense the polymer, leading to the ordered mesoporous carbon product. In a typical preparation, 2.26 g of formaldehyde (37 wt % aqueous solution) and 2.20 g of resorcinol are stirred together for 1 h. 2 mL of 0.1 M aqueous NH_4_OH is added and the solution stirred for 1 h, giving rise to the oligomer of resorcinol and formaldehyde, the “Resol”. This is added to a solution of Pluronic F127 (1.60 g) in ethanol (10 g) and distilled water (8 g), and the resultant solution is stirred for 20 min. The condensation catalyst, oxalic acid (0.225 g), is added, and after further stirring for 5–10 min, the solution becomes cloudy. The mixture is stirred for 1 h more and then is left to stand for 12 h until the polymer gel is obtained. After drying (room temperature, 12 h), it is cured (80 °C, 24 h) and then calcined in a tube furnace (400 °C, heating rate 1 °C·min^−1^) under flowing N_2_ (100 mL·min^−1^ STP) for 3 h to produce the OMC product as a fine, black powder.

### 3.3. Materials Characterisation

The OMC material was characterised using Fourier-transform infrared spectroscopy, X-ray diffraction, scanning electron microscopy (SEM), transmission electron microscopy (TEM) with X-ray emission spectroscopy for chemical analysis (energy-dispersive spectroscopy), and nitrogen gas physisorption. FT-IR spectra were recorded on an IRAffinity-1S instrument (Shimadzu, Kyoto, Japan) operated through LabSolutions IR software (version 2.1). A background spectrum was recorded prior to each measurement. A PANanalytical Empyrean instrument (Malvern Panalytical, Malvern, UK) using Cu Kα radiation and operating in reflectance geometry was employed to obtain XRD diffraction patterns over the 2θ range, 10–90°. SEM was carried out using a JEOL JSM-IT200 (JEOL, Tokyo, Japan) instrument. TEM was carried out using a Titan Themis 200 keV (FEI, Eindhoven, The Netherlands) transmission and scanning transmission electron microscope (S/TEM). This sophisticated instrument is equipped with an X-FEG Schottky field emission gun and incorporates a spherical aberration corrector for enhanced imaging quality. Elemental maps were also obtained in the Titan instrument in STEM mode using the SuperX windowless energy-dispersive spectrometer (FEI, Eindhoven, The Netherlands) supplied with the microscope. The samples for TEM analysis were prepared by ultrasonicating an OMC powder suspension in acetone for 1 min and capturing sample particles on a holey carbon-coated 300-mesh Cu grid by sweeping this through the suspension and then allowing the grid to dry under a halogen lamp overnight. Images were collected and analysed using Digital Micrograph software (version 3.51). A Micromeritics TriStar II 3020 instrument (Norcross, GA, USA) operating at liquid nitrogen temperature was used to obtain nitrogen physisorption isotherms (with samples degassed at 393 K for at least 12 h before measurement). The software supplied by the instrument manufacturer was used to retrieve the Barrett, Joyner, and Halenda (BJH) pore-size distribution data.

### 3.4. Instruments for Adsorption Experiments

M/s Merck, India, supplied the HCl and NaOH necessary for pH adjustments. The pH of the solutions was obtained using an Electronics India/ESICO digital pH meter (microprocessor-based, Pune, India). The dye was produced as a 0.01 M stock solution, and the studies were carried out using diluted solutions derived from it. The absorbance of the dye solutions was measured in batch processing using a UV–Vis Spectrophotometer (Esico, Parwanoo, India). For stirring, a water bath shaker was used (Remi RSB-12, Mumbai, India).

### 3.5. Adsorption Batch Experiments

To carry out batch adsorption, a known volume (20 mL) of the crystal violet dye solution (0.5 × 10^−5^ M to 5 × 10^−5^ M) is taken in a 100 mL stoppered volumetric flask at a fixed temperature, and the pH of this solution is maintained at a constant value (2–10). To this dye solution, a set proportion of adsorbent OMC (0.10 to 0.30 g) is added, and the mixture is agitated for a predetermined time interval (15 to 150 min) on a mechanical shaker coupled with a water bath. The solution is filtered, and the absorbance of the filtrate is recorded on a UV–Vis spectrophotometer at the maximum absorption wavelength of the dye (λ_max_ = 546 nm). Equation (17) was used to compute the adsorption capability at equilibrium:

(21) $$q_{e}=\frac{\left(C_{o}-C_{e}\right)\times \;V}{W}$$

where C_o_ is the pre-adsorption concentration and C_e_ is the dye’s equilibrium concentration in mol·L^−1^, V is the volume of the result admixture in litres, and w is the mass of the adsorbent used in g. For the preliminary investigation, variations in physico-chemical parameters are carried out, while kinetic and isothermal trials are carried out at optimum values of the parameters obtained during these preliminary investigations, following the same procedure.

Zero-point charge (pH_zpc_) is the pH of the medium at the zero electric potential over the adsorbent in its aqueous solution. In the present case, the pH_zpc_ of OMC was determined using the drift technique [65] by adding 0.1 g of OMC in 50 mL 0.1 M KCl solution, which is kept at various pH values (2 to 10). The suspension was stirred for 24 h of incubation and the change in pH (∆pH) is observed. The point at which ∆pH becomes zero is considered to be the pH_zpc_ of the OMC.

### 3.6. Fixed Bed Column Operations

#### 3.6.1. Dye Adsorption in OMC Fixed Bed Column

Fixed bed adsorption was carried out in a glass column of 20 cm length and 1 cm internal diameter. The adsorbent OMC (0.2 g) slurry was prepared in distilled water and kept overnight. A small amount of glass wool is placed in the bottom portion (about 1 cm) of the glass column, and the slurry is fed into the column. To avoid air entrapment in the column, the water is allowed to flow continuously out of the column. This resulted in a cylindrical packed adsorbent bed of about 0.9 cm in length.

After removing water from the column and keeping the adsorbent wet, it is loaded with a concentrated solution of the dye (5 × 10^−4^ M). The dye is allowed to percolate through the adsorbent bed at a flow rate of 4 mL/min. Aliquots of 10 mL each were collected and spectrophotometrically analysed at λ_max_ = 546 nm. When the concentration of collected aliquots and total dye solution fed to the column became equal, the operation was stopped. This is the stage when the adsorbent is thought to be completely exhausted (i.e., saturated by adsorbate molecules).

#### 3.6.2. Desorption of Column and Dye Retrieval

To recover the dye material dil. H_2_SO_4_ (0.1 M) is passed through the exhausted column at a constant flow rate of 5 mL·min^−1^. Spectrophotometric analysis is carried out for every 25 mL aliquot of the collected effluent. The process is continued till the concentration of the Crystal Violet dye reduces to nil. The effluent thus collected is then evaporated to dryness on a water bath and the dry material obtained was analysed by obtaining its FTIR spectrum, which matched with the spectrum of the original dye. This confirmed the retrieval of the same Crystal Violet dye material after its adsorption and desorption from the column.

#### 3.6.3. Determination of Column Efficiency

After the retrieval of Crystal Violet dye the column is thoroughly washed by percolating warm distilled water (about 300 mL) through it. When the collected effluent became colourless the column is considered to be completely washed and ready for the next column operation run to verify the column efficiency. Again, the same operation is repeated and the percentage saturation of the freshly available column was evaluated.

## 4. Conclusions

The OMC material synthesized from resorcinol and formaldehyde, and employing Pluronic F127 to provide a self-assembled micellar framework, was subjected to characterization using FT-IR, electron microscopy with elemental analysis (EDS), and N_2_ gas physisorption. The preparation method avoids the use of the more conventional NaOH and HCl polymerisation catalysts—employing rather NH_4_OH and oxalic acid—and therefore is free of metal and halide species. The material was found to have a very high specific surface area (568 m^2^g^−1^) and pore volume (0.585 cm^3^g^−1^), and to consist of uniform long, parallel cylindrical pores (~7.0 nm diameter) separated by thin pore walls. These contained around 70% C and 30% O, the presence of the latter being consistent with the detection of in ether, O–H and C–O bonds in FTIR. The chemical groups, when at the surface of the pores, are likely to assist in the adsorption of the Crystal Violet.

To verify the adsorption capabilities of the OMC towards the hazardous basic dye, Crystal Violet, preliminary adsorption investigations have been carried out under batch conditions by varying different physicochemical parameters. The optimum adsorption of the dye at 30 °C was obtained at a concentration of 5 × 10^−5^ M, pH 9, OMC dosage of 0.025 g and contact time of 120 min. Adsorption isotherm experiments were carried out to test against Langmuir, Freundlich, Temkin, and DR isotherm models and it is found that the Crystal Violet–OMC adsorption system follows all the four models. This indicates that dye ions adsorb over OMC through the chemisorption process forming a uniform monolayer, which later results into multilayer formation. With the help of Langmuir constants “b”, it is ascertained that adsorption of crystal violet on OMC is a feasible, spontaneous, endothermic process with increasing degree of disorder occurring during the process. Kinetic investigations reveal that the adsorption is a pseudo-second-order process involving film diffusion kinetics at all the temperatures.

Bulk removal of the dye was studied by percolating the dye solution through a fixed bed column of the OMC adsorbent and its saturation was calculated as 97%. The desorption of the dye from the exhausted column was carried out by using dil. H_2_SO_4_ and about 99% dye recovery was achieved. The column efficiency was determined by performing ten adsorption/desorption cycles and it is found that the dye recovery decreases to approximately 92% in the 10th cycle.

On the basis of the above results, we can safely state that amongst all the available adsorbents tried so far by different workers, OMC is the best and most efficient material for adsorbing crystal violet dye.

This study shows that the chemical and textural properties of OMC materials make them an interesting option for the removal of toxic dyes from wastewater. Of particular importance, with a view to their eventual industrial application, is the excellent cyclability demonstrated in this work. In future studies, it would be interesting to vary synthesis parameters in order to modify the chemical nature of the surface of the OMC—and perhaps its structural properties, such as pore size—in order to further improve adsorption performance. Long-term studies of the use of OMC materials as adsorbents coupled with “before and after” characterization to determine the chemical and structural changes this might cause would also be valuable.

## Figures and Tables

**Table 1:** Fundamental properties of the dye, crystal violet.

Other names	Aniline violet, Basic violet 3, Baszol Violet 57L, Brilliant Violet 58, Hexamethyl-p-rosaniline chloride, Methylrosanilide chloride, Methyl Violet 10B, Methyl Violet 10BNS, Pyoktanin
Melting point	205 °C (401 F: 478 K)
Molecular weight	407.99 g·mol^−1^
IUPAC name	4-{Bis[4-(dimethylamino)phenyl]methylidene}-N,N-dimethylcyclohexa-2,5-dien-1-iminium chloride
Chemical formula	C_25_H_30_CIN_3_
Class	Triarylmethane
Colour	Blue Violet
Solubility in water	4 g/L at 25 °C

**Table 2:** Values of constants obtained during adsorption isotherm studies of the crystal violet–OMC System at the temperatures indicated.

Langmuir Adsorption Isotherm			
Parameter	Temperature (°C)		
	30	40	50
q_o_ (g·g^−1^)	2.15	1.75	0.73
b × 10^4^ (L·mol^−1^)	1.85	2.27	3.62
r	0.52	0.47	0.36
R^2^	0.99	0.99	1.00
**Freundlich Adsorption Isotherm**			
$k_{f}$ × 10^4^ (mol·g^−1^)	1.11	5.67	2.06
n	0.94	0.83	0.88
R^2^	1.00	0.99	0.99
**Temkin Adsorption Isotherm**			
$k_{1}$ × 10^−1^ (mol·g^−1^)	2.30	2.42	2.15
$k_{2}$ × 10^5^ (L·mol^−1^)	5.79	5.32	4.99
R^2^	0.96	0.95	0.92
**Dubinin–Radushkevtich Adsorption Isotherm**			
X_m_ (mol·g^−1^)	1.34	3.01	1.82
β × 10^−9^ (L·mol^−1^)	7.00	7.00	6.00
E (kJ·mol^−1^)	8.45	8.45	9.12
R^2^	1.00	1.00	0.99

**Table 3:** Thermodynamic data for the uptake of crystal violet by OMC.

Parameters	Temperature (°C)		
	30	40	50
−ΔG° (kJ·mol^−1^)	24.76	26.11	28.19
ΔH° (kJ·mol^−1^)	16.12	27.31	39.23
ΔS° (kJ·K^−1^mol^−1^)	16.20	27.39	39.32

**Table 4:** Comparison of adsorption performance of OMC with other adsorbents for crystal violet dye adsorption under batch conditions.

Adsorbent	Maximum Adsorption Capacity (mg/g)	Optimal Adsorption Conditions				Ref.
		pH	Dose (g/L)	Time (h)	Conc.	
OMC	2150.00	9	1.0	2.0	4 × 10^−5^ M	Present Study
Bentonite–Alginate Composite	601.93	8	10.0	-	300 ppm	[49]
Water Hyacinth	322.58	7.8	1.5	2.0	100 ppm	[50]
Bio-Nanocomposite (Alg-Cst/Kal)	169.49	8	0.4	3.0	-	[51]
Modified Rice Husk	90.02	10	1.0	1.0	1000 ppm	[52]
Alligator weed Laminaria Japonica	82.83	10	-	2.0	5 g/L	[53]
Palm Kernel Fiber	78.9	7.2	0.15	1.5	-	[54]
Coniferous Pinus Bark Powder	32.78	8	1.0	2.0	50 ppm	[55]
Clay	25.98	10	1.0	0.25	30 ppm	[56]
TLAC/Chitosan Composite	12.5	9	4.0	0.67	-	[57]
Iron Based Metal Organic Framework	9.259	6	3.5	24.0	5 ppm	[58]
De-Oiled Soya	1.42	8	2.0	4.0.	8 × 10^−5^ M	[59]
Crosslinked Grafted Xanthan Gum	0.75	7	0.8	7.0	500 ppm	[60]
Bottom Ash	0.27	8	2.0	4.0	8 × 10^−5^ M	[59]

Note: All the crystal violet–adsorbent systems tabulated above follow pseudo-second-order kinetics, and all these processes are spontaneous, endothermic, and observe increasing disorder.

**Table 5:** Values of variable determined from calculations for the crystal violet adsorbed in OMC fixed bed column experiments.

C_o_ (M)	C_x_ (M)	C_b_ (M)	V_x_ (mL)	V_b_ (mL)	(V_x_ − V_b_) (mL)	F_m_ (mg/cm^2^/min)	D (cm)
5 × 10^−4^	4.94 × 10^−5^	2.96 × 10^−5^	400	40	85	1.167	0.90

**Table 6:** Parameters for adsorption of crystal violet dye on OMC fixed bed.

t_x_ (min)	t_δ_ (min)	t_f_ (min)	f	δ (cm)	Percentage Saturation
342.720	308.448	8.889	0.991	0.924	96.89

## Data Availability

Data are contained within the article.
